# 1,3-Dichlorobenzene – Evaluation of a biological guidance value (BLW): Assessment Values in Biological Material – Translation of the German version from 2026

**DOI:** 10.34865/bb54173e11_1or

**Published:** 2026-03-30

**Authors:** Andrea Kaifie, Wobbeke Weistenhöfer, Thomas Kraus, Hans Drexler, Andrea Hartwig, MAK Commission

**Affiliations:** 1 Friedrich-Alexander-Universität Erlangen-Nürnberg, Institute and Outpatient Clinic of Occupational, Social, and Environmental Medicine Henkestraße 9–11 91054 Erlangen Germany; 2 Institute for Occupational, Social and Environmental Medicine, Uniklinik RWTH Pauwelsstraße 30 52074 Aachen Germany; 3 Institute of Applied Biosciences, Department of Food Chemistry and Toxicology, Karlsruhe Institute of Technology (KIT) Adenauerring 20a, Building 50.41 76131 Karlsruhe Germany; 4 Permanent Senate Commission for the Investigation of Health Hazards of Chemical Compounds in the Work Area, Deutsche Forschungsgemeinschaft, Kennedyallee 40, 53175 Bonn, Germany. Further information: Permanent Senate Commission for the Investigation of Health Hazards of Chemical Compounds in the Work Area | DFG

**Keywords:** 1,3-dichlorobenzene, 3,5-dichlorocatechol, biological guidance value, BLW

## Abstract

In 2025, the German Commission for the Investigation of Health Hazards of Chemical Compounds in the Work Area has evaluated a biological guidance value (BLW) for 1,3‑dichlorobenzene [541-73-1]. Relevant publications were identified from a literature search. For the derivation of the BLW, an experimental study was available that demonstrated a good correlation between the concentration of 1,3-dichlorobenzene in the air and 1,3-dichlorobenzene in blood as well as the urinary metabolites 3,5‑dichlorocatechol, 2,4‑dichlorophenol and 3,5-dichlorophenol. The data of the experimental study conducted with resting volunteers for 6-hours were extrapolated to account for an exposure of 8 hours and an increased respiratory volume at the work place. At the level of the MAK value of 2 ml/m^3^, an average value of 40 mg 3,5-dichlorocatechol (after hydrolysis)/g creatinine can be expected and was set as BLW. Sampling time is at the end of the shift, after several previous shifts.

**Table:** 

**BLW (2025)**	**40 mg 3,5-dichlorocatechol (after hydrolysis)/g creatinine**
	Sampling time: at the end of the shift, for long-term exposures after several previous shifts
Synonyms	*m*-dichlorobenzene
Formula	C_6_H_4_Cl_2_
Molar mass	147 g/mol (IFA 2026)
Melting point	–22 °C (IFA 2026)
Boiling point	173 °C (IFA 2026)
Vapour pressure at 20 °C	2.4 hPa (IFA 2026)
Density at 20 °C	1.29 g/cm^3^ (IFA 2026)
log K_OW_	3.52 (IFA 2026)
Water solubility at 60 °C	0.201 g/l (IFA 2026)
	
**MAK value (2007)**	**2 ml/m^3^** **≙** **12 mg/m^3^**
Absorption through the skin	–
Carcinogenicity	–
Prenatal toxicity	Pregnancy Risk Group C

1,3-Dichlorobenzene is a colourless liquid with a pungent odour that forms as a degradation product in the manufacture of silicone rubber when bis(2,4-dichlorobenzoyl) peroxide (2,4-DCBP) is used as a radical initiator (Herkert et al. 2018).

## Metabolism and Toxicokinetics

**Fig. 1:**
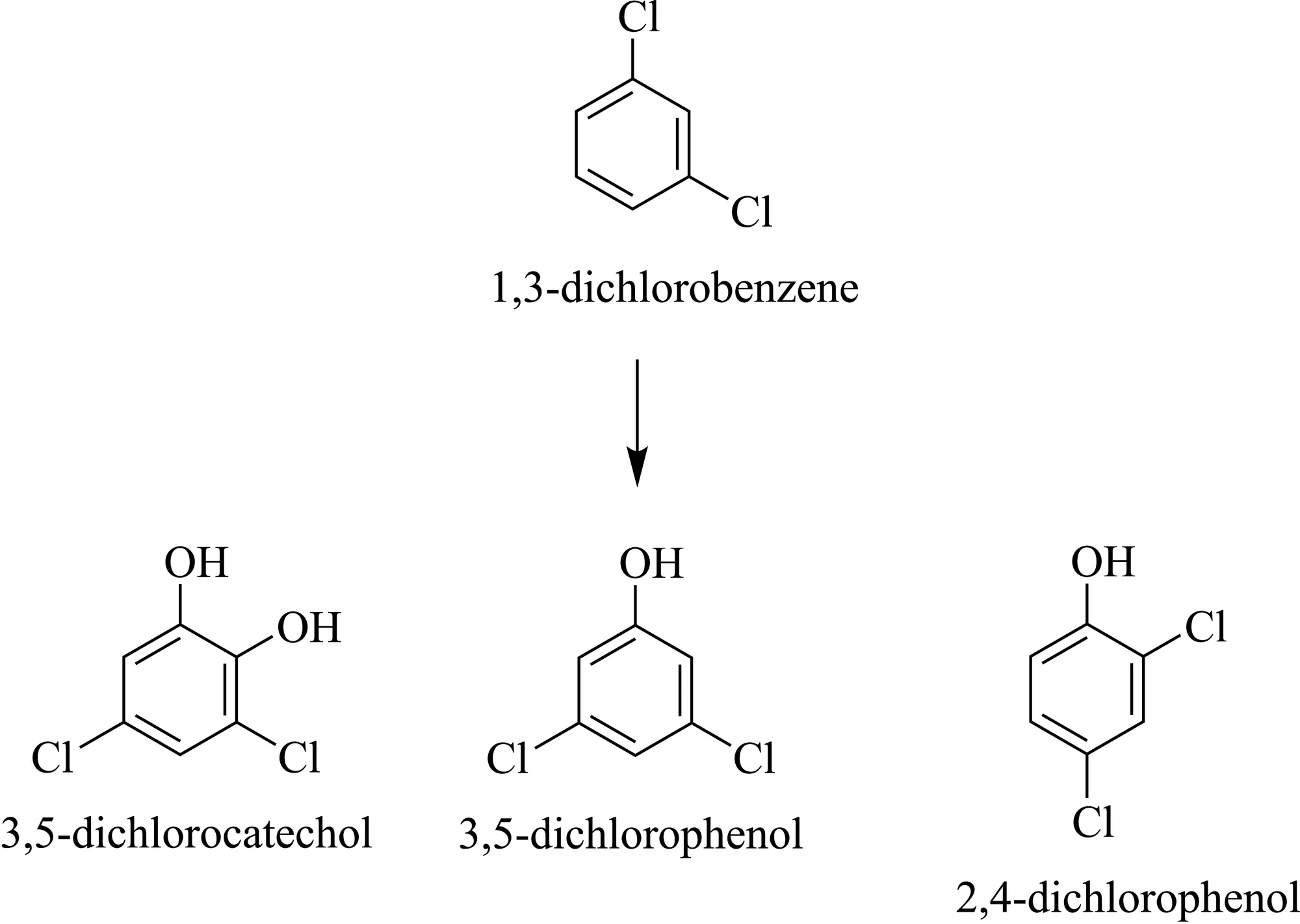
Phenolic metabolites of 1,3-dichlorobenzene in human urine

1,3-Dichlorobenzene is metabolised in the body to metabolites including 3,5-dichlorocatechol, 3,5-dichlorophenol and 2,4-dichlorophenol (Figure 1), which are excreted as glucuronide and sulfate with the urine.

Schettgen et al. (2022) investigated internal exposure to 1,3-dichlorobenzene and determined the metabolites 3,5-dichlorocatechol, 2,4-dichlorophenol and 3,5-dichlorophenol in the urine of 104 employees (82 men and 22 women) of a company that manufactures silicone rubber and uses 2,4-DCBP as a radical initiator. The three metabolites were detected in more than 89% of all urine samples. The maximum concentrations were 26.9 mg 3,5-dichlorocatechol/l, 7.68 mg 2,4-dichlorophenol/l and 0.39 mg 3,5-dichlorophenol/l urine.

## Critical Toxicity

No data on toxicity in humans are available.

The most sensitive effect after exposure to 1,3-dichlorobenzene is the induction of xenobiotic-metabolising enzymes, including glucuronosyl transferase I, in a 28-day study with gavage administration in rats, in which a LOEL (lowest observed effect level) of 4 mg/kg body weight and day was obtained (Hoechst AG 1989). It can be assumed that, for this particularly sensitive end point, the short-term high exposure due to bolus administration contributed to the LOEL value. The induction of liver enzymes should be regarded as the trigger for the changes in the thyroid follicles of the rats, which occurred significantly more frequently at doses of 37 mg 1,3-dichlorobenzene/kg body weight and day (LOAEL = lowest observed adverse effect level) and above in a 90-day study (McCauley et al. 1995). The observed thyroid changes are relevant also for humans, but occur only at higher doses than in rats (Lewandowski et al. 2004), as humans have better protective mechanisms available due to their ability to store thyroid hormones. In the 90-day study in rats, no adverse effects on the thyroid gland were observed at 9 mg/kg body weight and day. A MAK value of 2 ml 1,3-dichlorobenzene/m^3^ (12 mg/m^3^) was derived from this NOAEL (no observed adverse effect level) in rats (MAK Value Documentation 2008 translated in Hartwig 2013). In a re-evaluation, a more accurate species extrapolation was used to calculate a corresponding air concentration at the workplace of 18 mg/m^3^ and the MAK value was confirmed (Hartwig 2012, available in German only), as the underlying NOAEL was obtained for epithelial thyroid changes in rats, and rats are considered to be more sensitive than humans with regard to thyroid effects.

## Exposure and Effects

Ten healthy male volunteers were exposed to 1,3-dichlorobenzene for a total of six hours in a workplace simulation facility, followed by biomonitoring of 1,3-dichlorobenzene in the blood and determination of the metabolites 3,5-dichlorocatechol (DCK), 2,4-dichlorophenol (DCP) and 3,5-dichlorophenol in urine (Schettgen et al. 2023). There were three exposure scenarios: 1.5 ml 1,3-dichlorobenzene/m^3^ (8 mg/m^3^), 0.7 ml/m^3^ (4 mg/m^3^) and 1.5 ml/m^3^ with respiratory protective mask for gases and vapours (3M, 4251+). The subjects were at rest during exposure. Effect parameters were not investigated.

### 1,3-Dichlorobenzene in the blood

**Fig. 2:**
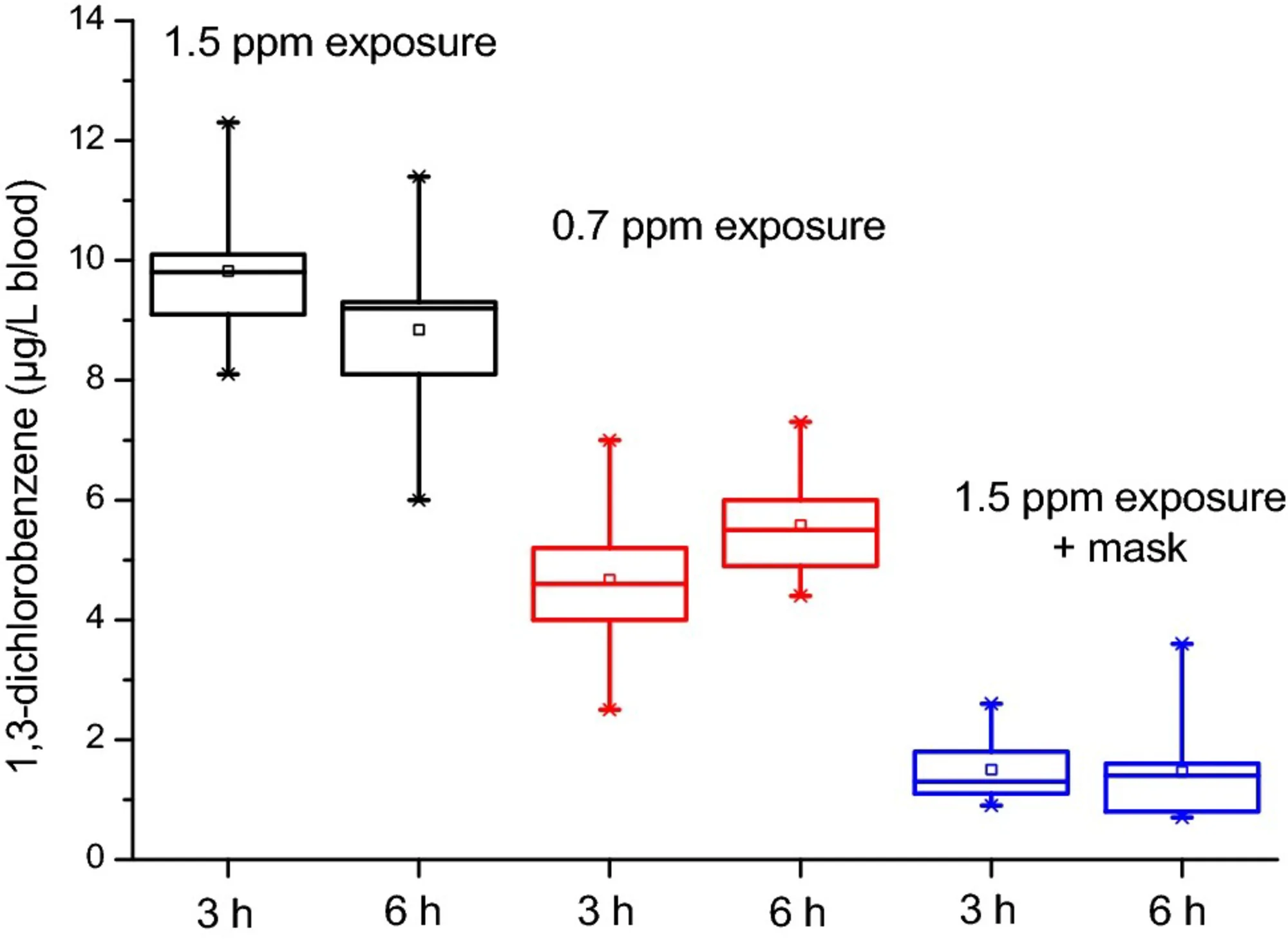
Concentration of 1,3-dichlorobenzene in blood after 3 and 6 hours in different exposure scenarios (Schettgen et al. 2023, published under a CC BY 4.0-License by Springer Nature)

Blood samples for the determination of 1,3-dichlorobenzene were taken three hours after the start of exposure and after six hours at the end of exposure. The concentration of 1,3-dichlorobenzene in the blood did not differ significantly between the sampling time-points. This indicates that a steady state has been reached after approximately three hours. When exposed to 1.5 ml 1,3-dichlorobenzene/m^3^, median 1,3-dichlorobenzene concentrations of 9.7 µg/l blood (after three hours) and 9.0 µg/l blood (after six hours) were determined (Figure 2).

The following regression equation (1) was derived for the relationship between 1,3-dichlorobenzene in the air and 1,3-dichlorobenzene in blood:

1,3-Dichlorobenzene [μg/l blood] = 5.26 × 1,3-dichlorobenzene [ml/m^3^ air] + 1.45 (1)

From this, a concentration of 12 µg 1,3-dichlorobenzene/l blood for an exposure at the level of the MAK value of 2 ml 1,3-dichlorobenzene/m^3^ is calculated.

### Metabolites in urine

Urine samples were collected before exposure (t_0_) and over a period of 24 hours. The highest concentration was found for 3,5-dichlorocatechol, followed by 2,4-dichlorophenol and 3,5-dichlorophenol. Under exposure to 1.5 ml 1,3-dichlorobenzene/m^3^, the maximum urine concentration for 3,5-dichlorocatechol (11 963 µg/g creatinine) was reached after an average of 6.6 hours. The maximum values for 2,4-dichlorophenol (3515 µg/g creatinine) and 3,5-dichlorophenol (173 µg/g creatinine) were determined after 6.4 hours. The half-lives for the metabolites were 9.7 hours for 3,5-dichlorocatechol, 5.8 hours for 2,4-dichlorophenol and 4.8 hours for 3,5-dichlorophenol.

The following regression equations (2–4) were derived for the relationship between 1,3-dichlorobenzene in the air and 3,5-dichlorocatechol, 2,4-dichlorophenol and 3,5-dichlorophenol in urine for an exposure period of 6 hours:

3,5-Dichlorocatechol [μg/g creatinine] = 8472 × 1,3-dichlorobenzene [ml/m^3^ air] –744 (2)

2,4-Dichlorophenol [μg/g creatinine] = 2452 × 1,3-dichlorobenzene [ml/m^3^ air] –163 (3)

3,5-Dichlorophenol [μg/g creatinine] = 128.4 × 1,3-dichlorobenzene [ml/m^3^ air] –20 (4)

Extrapolated to an exposure period of 8 hours, Schettgen et al. (2023) reported the following metabolite concentrations in urine (after hydrolysis) for exposure at the level of the MAK value of 2 ml 1,3-dichlorobenzene/m^3^: 3,5-dichlorocatechol: 21 600 μg/g creatinine, 2,4-dichlorophenol: 6300 μg/g creatinine and 3,5-dichlorophenol: 320 μg/g creatinine.

The variability of litre-based concentrations was greater (r = 0.6 and smaller) than that of creatinine-based concentrations. The creatinine concentration in the volunteers’ urine was in the range from 0.12 to 5.59 g creatinine/l; some of the values (n = 38 of 203 urine samples, approx. 19%) were therefore outside the recommended range of 0.3–3 g creatinine/l of urine. The values were nevertheless included in the analysis. One volunteer provided only three urine samples over 24 hours.

## Selection of the Indicators

Biomonitoring of persons exposed to 1,3-dichlorobenzene can be carried out by determining the concentration of 1,3-dichlorobenzene in whole blood.

The following metabolites of 1,3-dichlorobenzene are available in urine: 3,5-dichlorocatechol, 2,4-dichlorophenol, 3,5-dichlorophenol and the two isomers 2,4-dichlorophenyl mercapturic acid and 3,5-dichlorophenyl mercapturic acid.

## Test Methods

To determine 1,3-dichlorobenzene in whole blood, headspace gas chromatography mass spectrometry (GC/MS) is used, and for the metabolites 3,5-dichlorocatechol, 2,4-dichlorophenol and 3,5-dichlorophenol in urine, a fully automated solid-phase extraction (SPE) combined with liquid chromatography tandem mass spectrometry (LC-MS/MS) after hydrolysis is used as the test method (Schettgen et al. 2023). An online SPE LC-MS/MS method is available for determining mercapturic acids in urine (Schettgen et al. 2024).

## Background Exposure

There are no data available on background exposure to 1,3-dichlorobenzene or its metabolites in the general working-age population in Germany.

In a representative sample of the general population (age group ≥ 18 years) in the USA (National Health and Nutrition Examination Survey (NHANES)), the reference value was below the detection limit of 0.025 ng 1,3-dichlorobenzene/ml blood during the 2015/2016 survey period (CDC 2025).

## Evaluation of a Biological Guidance Value (BLW)

Only the study by Schettgen et al. (2023) is available for evaluating a limit value. The following limitations of the study must be taken into account:

- There was no physical activity during exposure to 1,3-dichlorobenzene. As the blood:air partition coefficient for the chemically similar 1,4-dichlorobenzene is 87.6, a factor of 2 is included in the calculation of the BLW to take account of the increased respiratory volume at the workplace.
- The study was conducted in only 10 people.
- In almost 20% of the volunteers, the creatinine concentration in urine was outside the recommended range of 0.3–3 g/l urine (WHO 1996), which limits the significance of their urine samples.
- Exposure took place over 6 hours instead of 8 hours with a half-hour break (2 × 3 hours).
- The exposure level of 1.5 ml 1,3-dichlorobenzene/m^3^ was below the level of the MAK value of 2 ml 1,3-dichlorobenzene/m^3^.

In view of the uncertainties mentioned above, a BLW is set for the metabolite 3,5-dichlorocatechol in urine, which showed the highest concentration of the parameters. Taking into account a factor of 2 for the increased respiratory volume at the workplace and after an extrapolation of a 6-hour exposure to an 8-hour exposure,

**a BLW of 40 mg 3,5-dichlorocatechol (after hydrolysis)/g creatinine is derived**

in correlation with the MAK value of 2 ml 1,3-dichlorobenzene/m^3^.

Since the half-life of 3,5-dichlorocatechol is 9.7 hours, metabolites may accumulate in the urine over several shifts. Therefore, sampling should be carried out at the end of the shift, for long-term exposures, after several previous shifts.

## Interpretation of Data

The BLW for 3,5-dichlorocatechol relates to normally concentrated urine, in which the creatinine concentration should be in the range of 0.3–3 g/l. As a rule, where urine samples are outside the above limits, a repetition of the measurement in normally hydrated test persons is recommended (translated in Bader et al. 2016).
